# Impact of conversion surgery after chemotherapy in patients with initially unresectable and recurrent biliary tract cancer

**DOI:** 10.1002/ags3.12713

**Published:** 2023-07-19

**Authors:** Ikuo Nakamura, Etsuro Hatano, Hideo Baba, Keiko Kamei, Hiroshi Wada, Junzo Shimizu, Masashi Kanai, Kenichi Yoshimura, Hiroaki Nagano, Tatsuya Ioka

**Affiliations:** ^1^ Department of Gastroenterological Surgery Hyogo Medical University Hyogo Japan; ^2^ Department of Surgery, Graduate School of Medicine Kyoto University Kyoto Japan; ^3^ Department of Gastroenterological Surgery, Graduate School of Medical Science Kumamoto University Kumamoto Japan; ^4^ Department of Surgery Kindai University Faculty of Medicine Osakasayama Japan; ^5^ Department of Gastroenterological Surgery Osaka International Cancer Institute Osaka Japan; ^6^ Department of Surgery Toyonaka Municipal Hospital Toyonaka Japan; ^7^ Department of Clinical Oncology and Pharmacogenomics, Graduate School of Medicine Kyoto University Kyoto Japan; ^8^ Center for Integrated Medical Research Hiroshima University Hiroshima Japan; ^9^ Department of Gastroenterological, Breast and Endocrine Surgery, Graduate School of Medicine Yamaguchi University Yamaguchi Japan; ^10^ Oncology Center Yamaguchi University Hospital Ube Japan

**Keywords:** biliary tract cancer, chemotherapy, cisplatin, conversion surgery, gemcitabine, S‐1

## Abstract

**Purpose:**

Gemcitabine, cisplatin, and S‐1 chemotherapy was superior to gemcitabine and cisplatin chemotherapy for progression‐free survival and overall survival for unresectable and recurrent biliary tract cancer in a randomized phase III trial (KHBO1401). This study aimed to evaluate the outcome of conversion surgery after chemotherapy in biliary tract cancer patients (ancillary study, KHBO1401‐3C).

**Methods:**

A total of 246 patients were enrolled in KHBO1401. We compared progression‐free and overall survivals between the conversion surgery and non‐conversion surgery groups.

**Results:**

Eight patients (3.3%) underwent conversion surgery with chemotherapy, seven of whom were diagnosed with unresectable disease and one with recurrence. Six and two patients received gemcitabine, cisplatin, and S‐1 chemotherapy as well as gemcitabine and cisplatin chemotherapy, respectively. Three patients in the conversion surgery group who received gemcitabine, cisplatin, and S‐1 chemotherapy showed no disease progression and survived without postoperative chemotherapy. Preoperative carbohydrate antigen 19‐9 (CA19‐9) level was a prognostic factor for conversion surgery. After correcting for immortal time bias, 1‐year progression‐free survival rates in the conversion surgery and non‐conversion surgery groups were 50.0% and 19.0%, respectively (hazard ratio 0.343, 95% confidence interval 0.286–0.843, *p* = 0.0092). One‐year overall survival rates in the conversion surgery and non‐conversion surgery groups were 87.5% and 56.0%, respectively (hazard ratio 0.222, 95% confidence interval 0.226–0.877, *p* = 0.0197).

**Conclusions:**

Conversion surgery might be an option for the treatment of unresectable and recurrent biliary tract cancer in patients with normal preoperative CA19‐9 level.

## Graphical abstract

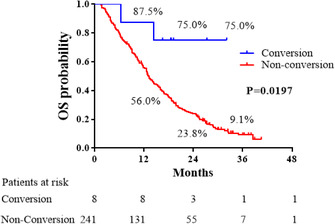

## 1 INTRODUCTION

In patients with unresectable cancer at the time of diagnosis, chemotherapy is the main treatment strategy. When the disease is controllable after chemotherapy, conversion surgery is performed for various cancers such as tumors that were initially deemed unresectable or marginally resectable due to technical and/or oncological reasonss.^1^, ^2^, ^3^ Several studies have reported that 12%–36% of patients with unresectable colorectal liver metastasis show sufficient response to chemotherapy and can undergo conversion surgery.^4^, ^5^, ^6^, ^7^, ^8^ Additionally, a recent retrospective global study on unresectable gastric cancer (CONVO‐GC‐1) reported 1206 patients with stage IV gastric cancer who had conversion surgery after chemotherapy, and the median survival time was 36.7 months.^9^

Biliary tract cancers (BTCs), including intrahepatic cholangiocarcinoma, extrahepatic bile duct cancer (ECC), gallbladder cancer (GBC), and ampulla of Vater cancer, are among the most refractory malignancies worldwide.^10^ Many patients are diagnosed at an advanced stage and cannot undergo curative resection. Although curative resection may be the only option for potentially curative treatment, the 5‐year overall survival (OS) rate postoperatively was 33.1% for ECC, 41.6% for GBC, and 52.8% for ampulla of Vater cancer.^11^ Therefore, systemic chemotherapy plays an important role in improving the outcomes of patients with BTCs. Recently, a randomized phase III study assessed the supremacy of combination therapy with gemcitabine, cisplatin, and S‐1 (GCS) over gemcitabine and cisplatin (GC) chemotherapy from the viewpoint of survival of patients with unresectable or recurrent BTC (KHBO1401).^12^ This study showed a high response rate (RR) in the GCS group (41.5%) compared with the GC group (15.0%) and significant survival advantage of GCS chemotherapy over GC chemotherapy. GCS chemotherapy has been shown to be a new standard treatment for patients with advanced BTC.

Conversion surgery after systemic chemotherapy for unresectable BTC has been separately described in several case reports.^13^, ^14^, ^15^, ^16^ However, the outcome of conversion surgery for unresectable and recurrent BTC remains unknown. Therefore, in this study, we evaluated the outcomes of patients who underwent conversion surgery after GC or GCS chemotherapy for unresectable and recurrent BTC in a randomized phase III study (KHBO1401) and investigated the impact of conversion surgery on unresectable and recurrent BTC.

## 2 METHODS

### 2.1 Patients and study design

**FIGURE 1:**
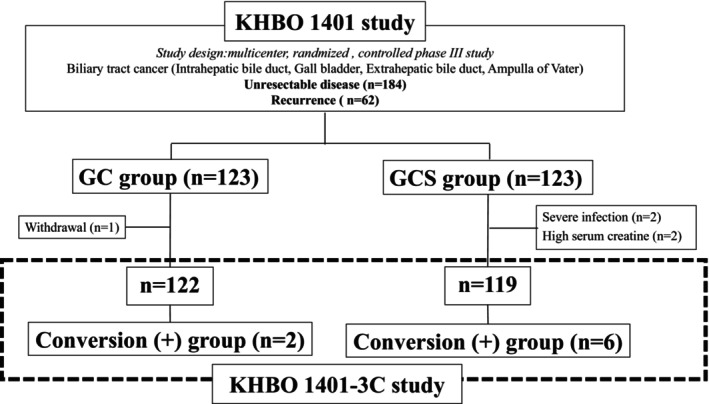
A schematic illustration of the study design (KHBO1401, ClinicalTrials.gov NCT02182778, and the UMIN Clinical Trials Registry, ID 000014371). The patients were divided into two groups (GC and GCS groups). Eight patients (two in the GC group and six in the GCS group) underwent conversion surgery and were investigated in the KHBO1401‐3C study. GC, gemcitabine and cisplatin; GCS, gemcitabine, cisplatin, and S‐1; KHBO, Kansai Hepatobiliary Oncology Group.

This study's design is shown in Figure 1. The KHBO1401 study was conducted by the Kansai Hepato‐Biliary Oncology Group (KHBO), wherein the eligibility criteria comprised chemotherapy‐naïve patients with advanced biliary tract adenocarcinoma (intrahepatic bile duct, gallbladder, extrahepatic bile duct, or ampulla of Vater), adequate organ function, and performance status of 0–2.^12^ The KHBO1401 study was enrolled with ClinicalTrials.gov (NCT02182778) and the UMIN Clinical Trials Registry (ID 000014371). The present 1401‐3C study is an ancillary study of KHBO1401.

From July 2014 to February 2016, 246 patients were registered in the KHBO1401 study. A total of 123 patients who received GC chemotherapy and 123 patients who received GCS chemotherapy had been allocated randomly to each group. Eight patients who underwent conversion surgery were enrolled in the KHBO1401‐3C study.

The study protocols of KHBO1401 and KHBO1401‐3C were approved by a suitably constituted Ethics Committee of each participating institution and conform to the provisions of the Declaration of Helsinki; Committee of Hyogo Medical University, Approval No 202204‐251. Before the study began, written informed consent was obtained from all the patients in KHBO1401 study.

### 2.2 Treatment in the KHBO1401 study

In the KHBO1401 study, two groups were defined: GC and GCS. The data center randomly assigned the patients to each group. In the GC group, gemcitabine and cisplatin were administered intravenously at doses of 1000 and 25 mg/m^2^, respectively, on days 1 and 8 every 3 weeks. In the GCS group, gemcitabine and cisplatin were administered intravenously at doses of 1000 and 25 mg/m^2^, respectively, on day 1, and oral S‐1 was administered daily at a dose of 80 mg/m^2^ on days 1–7 every 2 weeks. This is the regular dose for unresectable and recurrent BTCs and the recommended dose by the KHBO1002 study.^17^ We complied with the protocol of the KHBO1401 study reported previously.^12^

### 2.3 Pretreatment and follow‐up evaluation in the KHBO1401 study

Pretreatment evaluation was performed according to the protocol of the KHBO1401 study reported previously.^12^ Carcinoembryonic antigen (CEA) and carbohydrate antigen 19‐9 (CA19‐9) levels were measured at enrollment and monthly thereafter.^12^ Pathological data included T and N status according to the TNM Classification of Malignant Tumors, 7th edition, by Union for International Cancer Control.^18^

Imaging tests were performed 12 weeks after the initiation of treatment. Additional imaging tests were performed if required clinically or at the discretion of the treating physician. In patients with measurable target lesions, the objective RR was assessed according to the Response Evaluation Criteria in Solid Tumors (RECIST) version 1.1.^19^

### 2.4 Conversion surgery

Imaging tests were regularly planned during chemotherapy. Conversion surgery was performed in patients with adequate reduction in the main tumor, enabling complete removal inclusive of major vessels and metastatic sites; those with at least several months of local control; those with no new metastasis; or those with controllable metastasis by resection. Postoperative chemotherapy was performed after the conversion surgery if deemed necessary by the physician.

The extent of conversion surgery was decided according to the extent of disease progression, liver function status, and general condition of the patient. Liver function was evaluated by liver biochemistry tests, Child–Pugh classification, and measurement of the indocyanine green retention after 15 min.

### 2.5 Outcomes

The primary endpoints were progression‐free survival (PFS) and OS. PFS was defined as the time from the date of registration to disease progression after conversion surgery or death from any cause, and OS was defined as the time from the date of registration to death from any cause. In the conversion group (C group), when immortal time bias was considered, PFS or OS were calculated as the interval between the operation date and disease progression or death, or between the operation date and death. Depth of response, which was defined as the maximum tumor shrinkage rate in patients with target lesion, was also evaluated. Postoperative complications were classified according to the Clavien–Dindo classification.^20^ Postoperative mortality was defined as death during the postoperative hospital stay or within 90 days of the operation.

### 2.6 Statistical analysis

Continuous data are shown as median (range) or mean ± standard error of the mean (SEM). The Mann–Whitney U test or chi‐square test was performed to compare patient characteristics between the two groups. All factors were analyzed by univariate analyses.

Progression‐free survival and OS rates were analyzed using the Kaplan–Meier test and compared using the log‐rank tests. The comparison of PFS and OS between the C group and non‐conversion group (NC group) was performed with and without considering the immortal time bias. While considering the immortal‐time bias, sensitivity analysis was performed to underestimate the survival time of the conversion arm as a worst‐case scenario. Patients who had conversion surgery must survive at least until the date of the operation, or death cannot occur until the operation day for those who had conversion surgery; this is called “immortal time bias,” which means that there is an interval during which the study event cannot occur.^21^ Thus, in order for OS analysis to correct the potential immortal time bias due to patients who had conversion surgery, the operation status was considered a time‐varying covariate. In detail, eight patients in the C group were added to the NC group with censoring when they had the operation. PFS in the C group was calculated as the interval between the operation date and disease progression or death after the operation and OS as the interval between the operation date and death after the operation.

A two‐sided *p* value of <0.05 was defined as statistically significant. All statistical analyses were done using SPSS software (version 22; IBM Corp.). All data were analyzed under the statistician's supervision (K.Y).

## 3 RESULTS

### 3.1 Background of patients with conversion surgery

The trial profile is shown in Figure 1. Of the 246 patients who were enrolled in KHBO1401, 184 (74.8%) and 62 (25.2%) patients were diagnosed with unresectable disease or had recurrence, respectively, and eight (3.3%) underwent conversion surgery after tumor reduction with chemotherapy. The characteristics of these eight patients are summarized in Table 1. The median age was 67.5 years. Among them, three patients (37.5%) were male and five (62.5%) were female. The patients did not have hepatitis B or hepatitis C virus. Three patients (37.5%) had intrahepatic cholangiocarcinoma, two (25%) had hilar bile duct cancer, and two (25%) had GBC. Seven patients (87.5%) were diagnosed with unresectable disease and one (12.5%) had recurrence. All seven patients with an unresectable disease had distant metastases.

**TABLE 1:** Background of patients with conversion surgery.

Case no	Age (years)	Sex	Primary lesion	Disease status	UICC‐stage^a^	T	N	M	Distant metastasis	Target lesion	Histological differentiation
1	73	F	Perihilar	Unresectable	4B	T4	N1	M1	Liver (single), Lung (single)	−	Well
2	66	M	Perihilar	Unresectable	4B	T4	N1	M1	Bone (single), LN (Mediastinum, Left subclavian)	+	Undeterminable
3	68	F	Intrahepatic	Unresectable	4B	T3	N1	M1	LN (station16)	+	Undeterminable
4	69	F	Gall bladder	Unresectable	4B	T3	N1	M1	Lung (single), LN (station 16)	+	Poorly
5	53	F	Gall bladder	Unresectable	4B	T3	N1	M1	Liver (single), LN (station 16)	+	Undeterminable
6	67	M	Intrahepatic	Unresectable	4B	T3	N1	M1	Lung (multiple), LN (station 7)	+	Undeterminable
7	54	F	Intrahepatic	Unresectable	4B	T3	N1	M1	LN (station 16)	+	Moderately
8	80	M	Perihilar	Recurrent disease					−	+	Well

Abbreviations: F, female; LN, Lymph node; M, male; M, metastases; N, nodes; T, tumor; UICC, The Union for International Cancer Control.

^a^ TNM Classification of Malignant Tumors, 7th ed.

Of the eight patients, six (75%) received GCS chemotherapy and two (25%) received GC chemotherapy (Table 2). The number of cycles of protocol chemotherapy was 10 (1–12) (median, range). Four patients (50%) received chemotherapy after the protocol chemotherapy. The median (range) duration from registration to operation and from the last chemotherapy to operation was 9.0 months (6.0–19.0 months) and 1.7 months (0.5–4.4 months), respectively. The median (range) depth of target lesion response was 34.0% (18.0%–63.0%) in seven patients, except for one patient who had no target lesion. Specifically, the depth of target lesion response was 46.0% (29.0%–63.0%) in two patients with GC chemotherapy and 34.0% (18%–56.0%) in five patients with GCS chemotherapy. One of the patients had 63% of the highest reduction rate of LN station 8. In the evaluation of the target lesions before surgery, four patients showed partial response (PR) and three patients had stable disease (SD).

**TABLE 2:** Chemotherapy and operation data of patients with conversion surgery.

Case no	Type of protocol chemotherapy	Number of cycles of protocol chemotherapy	Next treatment after protocol chemotherapy	Duration from registration to operation (months)	Duration from the last chemotherapy to operation (months)	Depth of response of target lesion (%)	Evaluation of chemotherapy	The status of distant metastasis at operation	Progression‐free survival before the operation (months)	CEA negative conversion	Remaining normal level of CEA	Normal CEA level at the time of operation	CA19‐9 negative conversion	Remaining normal level of CA19‐9	Normal CA19‐9 level at the time of operation	Type of surgery	The resection of distant metastasis at operation
1	GCS	1	GS	11.2	1.4	No target lesion	No target lesion	Lung: CR, Liver: CR	9.9	−	+	+	−	−	−	Right hemihepatectomy + caudal lobectomy + extrahepatic bile duct resection + partial resection of portal vein	−
2	GCS	12	−	19	4.4	56	PR	Bone: CR, LN: CR	19.3	−	−	−	+	−	+	Left hemihepatectomy	−
3	GCS	12	−	7	2.3	36	PR	LN: SD	5.4	−	+	+	+	−	+	Right hemihepatectomy + caudal lobectomy + extrahepatic bile duct resection	LN
4	GCS	12	−	6	0.5	21	SD	Lung: SD, LN: SD	6.8	−	−	−	−	+	+	Hepatectomy of segment 4a and 5 + subtotal stomach‐preserving pancreatoduodenectomy	LN
5	GC	10	GC	12	1.8	63	PR	Liver: PR, LN: CR	13.1	+	−	+	−	+	+	Extended Left hemihepatectomy + extrahepatic bile duct resection	Liver
6	GC	8	GC	10	2.8	29	SD	Lung: CR, LN: SD	9.3	−	+	+	+	−	+	Left hemihepatectomy + extrahepatic bile duct resection	LN
7	GCS	10	−	7	1.7	18	SD	LN: PR	6.4	−	+	+	−	+	+	Anterior segmentectomy	LN
8	GCS	8	G	8	0.6	34	PR	−	6.7	−	+	+	−	+	+	Partial resection of segment 5	−

Abbreviations: CA19‐9, Carbohydrate Antigen 19‐9; CEA, Carcinoembryonic Antigen; CR, complete response; GC, gemcitabine and cisplatin combination therapy; GCS, gemcitabine, cisplatin, and S‐1 combination therapy; LN, lymph node; PR, partial response; SD, stable disease.

Among the seven patients with distant metastasis, there were three cases of lung metastasis, two cases of liver metastasis, one case of bone metastasis, and six cases of LN metastasis (Table 2). Two out of the three cases of lung metastasis achieved CR after chemotherapy and did not undergo resection during and after the conversion surgery. The remaining case (Case 4) was a solitary lesion with SD and did not undergo resection during the conversion surgery; resection after the conversion surgery was planned; however, it was not undertaken due to the postoperative development of new lesions in the lungs. One case of liver metastasis achieved CR and did not undergo resection during and after the conversion surgery, while the other achieved PR and was resected during the conversion surgery. The bone metastasis case achieved CR and did not undergo resection during and after the conversion surgery. Among the six cases of LN metastasis, two cases that achieved CR did not undergo resection during and after the conversion surgery. One case with PR and three cases with SD underwent resection during the conversion surgery.

Eight patients underwent liver resection, although one patient underwent subtotal stomach‐preserving pancreatoduodenectomy (SSPPD). In particular, Case 4 with GBC had LN metastasis (LN station 13a) behind the pancreatic head, which had invaded the head of the pancreas, and underwent hepatectomy of segments 4a and 5 as well as SSPPD. The liver function of the eight patients who underwent conversion surgery was good (data not shown). Only one patient received postoperative chemotherapy.

Surprisingly, three of the patients who received GCS chemotherapy preoperatively and did not undergo postoperative chemotherapy survived without disease progression after surgery. Of these patients, two were diagnosed with intrahepatic cholangiocarcinoma, whereas the other was diagnosed with hilar bile duct carcinoma. The other five patients had disease progression after the operation, two of whom died and the remaining three received treatment for disease progression. The median (range) follow‐up duration of the eight patients who underwent conversion surgery was 30 months (13–43 months).

### 3.2 Postoperative complications in patients with conversion surgery

According to the Clavien–Dindo classification of surgical complications, three patients (37.5%) had grade II, one (12.5%) had grade III, and one (12.5%) had grade IV complications (Table 3). There was no postoperative mortality after the conversion surgery.

**TABLE 3:** Perioperative outcomes in patients with conversion surgery.

Variables	Number of patients (%)
Duration of operation (min), median (range)	475 (193–799)
Bleeding volume (mL), median (range)	611 (140–2175)
Blood transfusion (present/absent)	3 (37.5)/5 (62.5)
Complication (present/absent)	5 (37.5)/3 (62.5)
Biliary leakage	2
Abdominal infection	2
Paralytic ileus	1
Clavien–Dindo classification	
I	0
II	3
IIIa	0
IIIb	1
IVa	0
IVb	1
V	0

### 3.3 Comparison of background between patients with and without conversion surgery

The rate of conversion surgery was 1.6% (two in 122 cases) in the GC group and 5.0% (six in 119 cases) in the GCS group, with no significant difference (*p* = 0.140) (Table 4). The RR (CR + PR) in the C group and NC group was 50.0% and 21.5% (*p* = 0.057), respectively. The C group had more patients with a normal CA19‐9 level at the time of the conversion surgery, that is, negative conversion of CA19‐9 or remaining normal level of CA19‐9, than the NC group (87.5% vs. 27.0%, *p* < 0.001). In the C group, more patients achieved preoperative PFS (6 months or more) than in the NC group (87.5% vs. 52.4%, *p* = 0.050). In the multivariate analysis, a normal CA19‐9 level at the conversion surgery was a prognostic factor (*p* = 0.019). The median depth of response in the C group was higher than that in the NC group (34% vs. 6%, *p* = 0.037) (data not shown). Some patients had no target lesion in both the C and NC groups. Therefore, the median depth of response was not included in the multivariate analysis.

**TABLE 4:** Comparison of background of patients with and without conversion surgery.

Variables	Univariate analysis			Multivariate analysis		
	Conversion (−)	Conversion (+)	*p*‐value	RR	95% CI	*p*‐value
Age (years), median (range)	68 (39–884)	67.5 (53–80)	0.755			
Sex			0.343			
Male	106 (45.5)	5 (62.5)				
Female	127 (54.5)	3 (37.5)				
Smoking			0.55			
Negative	141 (60.5)	4 (50.0)				
Positive	92 (39.5)	4 (50.0)				
Past medical history			0.764			
Negative	104 (44.6)	4 (50.0)				
Positive	129 (55.4)	4 (50.0)				
Obstructive jaundice			0.261			
Negative	128 (54.9)	6 (75.0)				
Positive	105 (45.1)	2 (25.0)				
Primary lesion			0.912			
Intrahepatic	74 (31.8)	3 (37.5)				
Gallbladder	80 (34.3)	2 (25.0)				
Extrahepatic	74 (31.8)	3 (37.5)				
Ampulla of Vater	50 (73.5)	0 (0.0)				
Gall bladder cancer			0.584			
Negative	153 (65.7)	6 (75.0)				
Positive	80 (34.3)	2 (25.0)				
Disease status			0.397			
Unresectable disease	173 (74.2)	7 (87.5)				
Recurrent disease	60 (75.8)	1 (12.5)				
Histological type			0.646			
Adenocarcinoma	227 (97.4)	6 (75.0)				
Adenosquamous cell carcinoma	6 (2.6)	2 (25.0)				
Histological differentiation			0.756			
Well	30 (12.9)	2 (25.0)				
Moderate	51 (21.9)	1 (12.5)				
Poor	28 (12.0)	1 (12.5)				
Unknown	124 (53.2)	4 (50.0)				
Chemotherapy			0.140			
GC	120 (51.5)	2 (25.6)				
GCS	113 (48.5)	6 (75.0)				
CR + PR			0.057	1.476	0.312–6.971	0.623
−	183 (78.5)	4 (50.0)				
+	50 (21.5)	4 (50.0)				
CR + PR + SD			0.397			
−	60 (25.8)	1 (12.5)				
+	173 (74.2)	7 (87.5)				
Normal CEA level at the time of operation			0.140			
−	120 (51.5)	2 (25.0)				
+	113 (48.5)	6 (75.0)				
Normal CA19‐9 level at the time of operation			<0.001	13.416	1.526–117.917	0.019
−	170 (70.3)	1 (12.5)				
+	63 (27.0)	7 (87.5)				
Progression‐free survival time before the operation (≧6 months)			0.050	2.793	0.288–27.102	0.376
−	111 (47.6)	1 (12.5)				
+	122 (52.4)	7 (87.5)				

Abbreviations: CA19‐9, Carbohydrate Antigen 19‐9; CEA, Carcinoembryonic Antigen; CR, complete response; GC, gemcitabine and cisplatin therapy; GCS, gemcitabine, cisplatin, and S‐1 combination therapy; PR, partial response; SD, stable disease.

### 3.4 Comparison of outcomes in patients with and without conversion surgery

**FIGURE 2:**
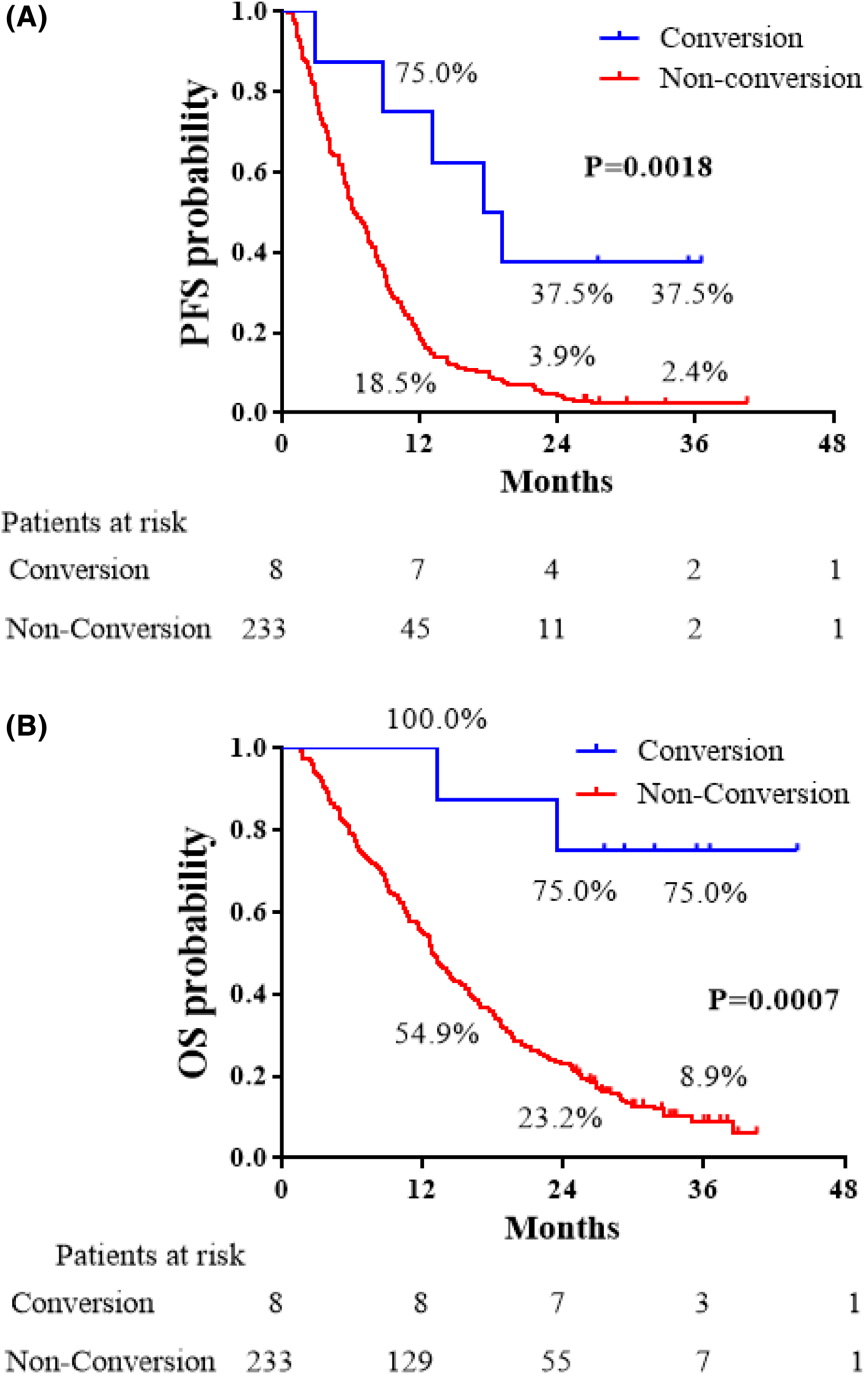
Progression‐free survival (PFS) and overall survival (OS) curves. PFS was defined as the time from the date of registration to disease progression after conversion surgery or death from any cause, and OS was defined as the time from the date of registration to death from any cause. The lines indicate the survival curves of patients with (blue line) and without (red line) conversion surgery. (A) PFS curves of patients with and without conversion surgery. (B) OS curves of patients with conversion surgery and without conversion surgery.

After the operation, five patients (62.5%) had a progressive status as a new lesion (Table 5). Six patients (75%) were alive at the end of the observation period. In the comparison between the two groups, the PFS and OS of the C group were superior to those of the NC group (*p* = 0.0018 and *p* = 0.0007, respectively) (Figure 2A,B). In the C group, the 1‐, 2‐, and 3‐year PFS rates were 75.0%, 37.5%, and 37.5%, respectively (hazard ratio [HR] 0.276, 95% confidence interval [CI] 0.274–0.731). In the NC group, the 1‐, 2‐, and 3‐year PFS rates were 18.5%, 3.9%, and 2.4%, respectively. The 1‐, 2‐, and 3‐year OS rates of the C group after registration were 100%, 75.0%, and 75.0%, respectively, whereas those of the NC group were 54.9%, 23.2%, and 8.9%, respectively (HR 0.132, 95% CI 0.226–0.663).

**TABLE 5:** Outcome data in patients with conversion surgery.

Case no	Postoperative chemotherapy	Progression‐free survival time after operation (months)	Status of progressive disease after operation	Organ of new lesion	Treatment of progressive disease	Alive or dead	Overall survival time after registration (months)	Overall survival time after operation (months)
1	S–1	21	New lesion	Lung	–	Alive	44	32.1
2	–	29	–	–	–	Alive	36.5	16.5
3	–	44.7	–	–	–	Alive	35.4	27.3
4	–	1.5	New lesion	Lung	–	Dead	13.2	6.4
5	–	6.3	New lesion	Peritoneum	Operation	Alive	31.8	18.5
6	–	9.7	New lesion	LN	S‐1 + RT	Alive	29.2	18.6
7	–	25.5	–	–	–	Alive	27.5	19.2
8	–	4.1	New lesion	LN	S‐1 + RT	Dead	23.5	14.3

Abbreviations: GS, gemcitabine and S‐1 combination therapy; RT, radiation therapy.

**FIGURE 3:**
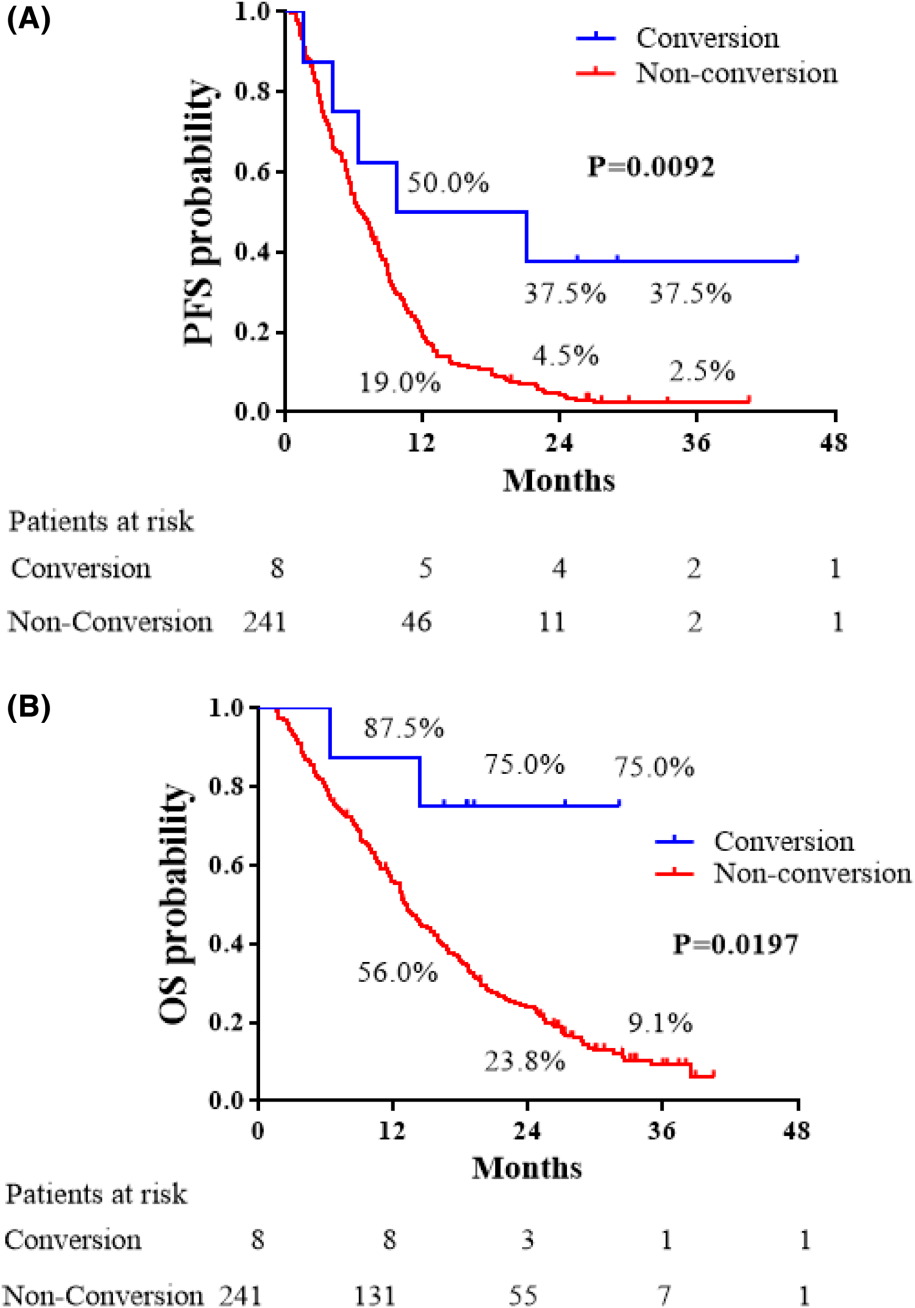
Progression‐free survival (PFS) and overall survival (OS) curves after correcting for immortal time bias using time‐varying covariate. The lines indicate the survival curves of patients with (blue line) and without (red line) conversion surgery. (A) PFS curves of patients with and without conversion surgery. (B) OS curves of patients with and without conversion surgery.

After correcting for immortal time bias, patients in the C group showed significantly improved PFS and OS compared with patients in the NC group (Figure 3A,B). One‐, 2‐, and 3‐year PFS rates after registration in the C group were 50.0%, 37.5%, and 37.5%, respectively. Those in the NC group were 19.0%, 4.5%, and 2.5%, respectively (HR 0.343, 95% CI 0.286–0.843, *p* = 0.0092). One‐, 2‐, and 3‐year OS rates after the operation in the C group were 87.5%, 75.0%, and 75.0%, respectively. Those in the NC group were 56.0%, 23.8%, and 9.1%, respectively (HR 0.222, 95% CI 0.226–0.877, *p* = 0.0197).

In a subgroup analysis, the 1‐year OS rate in two patients with GC chemotherapy was 100.0%, while the 1‐ and 2‐year OS rates in six patients with GCS chemotherapy were 83.4% and 66.7%, respectively, even after correcting for immortal time bias (Figure S1A,B). However, we did not compare patients with GC chemotherapy and those with GCS chemotherapy due to the limited number of patients in each group.

## 4 DISCUSSION

To our knowledge, this is the first study in which outcomes of conversion surgery after chemotherapy for unresectable and recurrent BTC were investigated. In this study, we investigated the PFS and OS of patients who underwent conversion surgery after chemotherapy for unresectable and recurrent BTC and showed the possibility that conversion surgery prolonged the survival of patients who had a high depth of target lesion response compared with patients without conversion surgery.

Several chemotherapy regimens for unresectable and recurrent BTC have different RRs. In the ABC‐02 study, the RR of GC therapy (vs. GEM alone) was 26.1% (vs. 15.5%).^22^ On the other hand, RR was 32.4% for GC and 29.8% for GS in the JCOG1113 study (FUGA‐BT).^23^ Based on these results, phase I and II studies on GCS therapy have been conducted.^17^, ^24^, ^25^, ^26^, ^27^, ^28^ Subsequently, a randomized phase III trial using our GCS regimen was conducted (KHBO1401 study) (NCT02182778, UMIN 000014371).^12^ In the KHBO1401 study, RR was 41.5% in the GCS arm and 15.0% in the GC arm. In this study, in the evaluation of chemotherapy before surgery, four patients showed PR, and three patients had SD. The RR in the C group was better than that in the NC group (50.0% vs. 21.5%, *p* = 0.057). In the retrospective analyses of clinical trial data, the depth of response was used as a measure for assessing tumor response.^29^, ^30^ The depth of response may be related to post‐progression survival. Sagawa et al.^31^ demonstrated that the depth of response can predict treatment outcomes in metastatic colorectal cancer patients treated with first‐line chemotherapy. In the current study, the univariate analysis showed that the depth of response in the C group was significantly higher than that in the NC group (Data not shown). Unfortunately, it was not used in the multivariate analysis because some patients had no target lesions in both the C and NC groups and the depth of response data were missing. Moreover, we could not show the relationship between the depth of response and postoperative outcomes because the number of patients who underwent conversion surgery was only eight. A larger prospective study will need to be conducted in the future to show this relationship. The optimal duration of preoperative therapy is debatable. Satoi et al.^32^ reported a significantly favorable OS in patients with initially unresectable pancreatic cancer who underwent conversion surgery more than 240 days after initial treatment, including gemcitabine‐based chemo(radio)therapy. In their study, the median duration between the initial therapy and PR/CR detection was 150 days and between PR/CR detection and the operation was 127 days. In metastatic pancreatic cancer, the median interval between diagnosis and conversion surgery was about 10 months.^33^, ^34^ In this study, the median preoperative PFS time was 8.1 (5.4–19.3) months in the C group. The C group had a longer preoperative PFS time (6 months or more) than the NC group with no significant difference; however, preoperative PFS time >6 months may be necessary. Tumor markers have evaluated the indication for surgery after chemotherapy in various types of cancer.^35^, ^36^ CEA uptrend was a better predictor of survival outcomes than conventional CEA measurements in patients undergoing hepatectomy for colorectal liver metastasis.^36^ In this study, a normal CA19‐9 level at the time of the operation was identified as a predictor for conversion surgery.

In the subgroup analysis, two GC therapy cases were compared with six GCS therapy cases. GC therapy showed a greater depth of response and favorable OS. However, limited sample size precludes statistical significance and warrants larger prospective studies for further investigation.

Unresectable BTCs are classified into BTCs with distant metastasis and locally advanced BTCs. In the former classification, if distant metastasis exhibits CR, PR, or SD and the primary lesion is resectable after chemotherapy by at least several months, then conversion surgery can be performed. In this study, distant metastases that achieved CR were observed without surgical removal (Cases 1 and 2). Conversely, distant metastases that achieved PR or SD were removed simultaneously during conversion surgery (Cases 3–7). In Case 4, LN metastasis was removed during the conversion surgery; the plan to remove the solitary lung metastasis after conversion surgery was canceled due to new lesions in the lungs postoperatively. Therefore, it is considered appropriate to observe distant metastatic lesions that achieve CR after chemotherapy without resection because they cannot be removed. For metastatic lesions with PR or SD, resection is recommended if the lesions are located within the surgical field and can be feasibly removed. However, if the metastatic lesions are distant from the surgical field, postoperative resection is recommended. Locally advanced unresectable BTCs are characterized by extensive tumor spread, involvement of blood vessels, or tumors with a size and localization that makes surgical intervention highly invasive and may result in a high risk of postoperative complications. However, if chemotherapy leads to shrinking effects, such as a reduction in tumor size or disappearance of vascular invasion, conversion surgery might be possible. Previous reports have discussed surgery after downstaging post‐chemotherapy or conversion surgery for locally advanced BTCs.^37^, ^38^, ^39^ In this study, all seven cases of inoperable BTC had distant metastasis, and there were no cases of locally advanced BTC.

While the efficacy of S‐1 as an adjuvant therapy for BTC has been demonstrated in the JCOG1202 trial,^40^ no reports indicate the efficacy of adjuvant chemotherapy after conversion surgery. In this study, adjuvant chemotherapy was administered after conversion surgery in Case 1; both lung and liver metastases achieved CR after chemotherapy in this case. The treating physician deemed a high risk of recurrence after surgery and opted for adjuvant chemotherapy. However, a new lung lesion was detected 21 months postoperatively. Among the seven cases that did not receive postoperative adjuvant chemotherapy, three did not experience recurrence. This is a retrospective study where the treatment after protocol therapy completion was left to the discretion of the treating physicians at each institution. Therefore, further research is needed to investigate adjuvant chemotherapy efficacy after conversion surgery.

In our latest prognosis survey for the eight cases of conversion surgery, the median follow‐up period was 39.2 (13.3–97.5) months. The survival rates were 100% at 1 year, 62.5% at 3 years, 31.3% at 5 years, and 15.6% at 7 years. The conversion surgery group showed significantly better prognosis than the NC surgery group, which had an MST of 12.9 months and survival rates of 54.9% at 1 year and 8.9% at 3 years.

This study has several limitations. First, the number of patients who underwent conversion surgery after chemotherapy was very small, and it was also difficult to perform a subgroup analysis, such as the comparison of GCS chemotherapy (*n* = 6) with GC chemotherapy (*n* = 2) in the conversion group. Further studies are thus necessary. Second, S‐1 has not been approved for use internationally. The pharmacokinetics and pharmacodynamics of S‐1 shows a difference between Caucasian and Asian patients.^41^, ^42^ Therefore, its safety and effectiveness in Caucasian patients will be monitored in further studies.

In conclusion, we reported cases of conversion surgery in the KHBO1401 study. The patients who underwent conversion surgery showed better outcomes compared with those who did not undergo conversion surgery. If patients have normal CA19‐9 level at the time of operation, conversion surgery might be a successful treatment option.

## AUTHOR CONTRIBUTIONS

Ikuo Nakamura contributed to literature search, study design, analysis plan, data analysis and interpretation, and drafting of article. Etsuro Hatano contributed to study design, data interpretation and analysis, and article revision. Hideo Baba, Keiko Kamei, Hiroshi Wada, and Junzo Shimizu contributed to acquisition of the data. Masahumi Kanai, Kenichi Yoshimura Hiroaki Nagano, and Tatsuya Ioka contributed to study design and data interpretation and analysis.

## FUNDING INFORMATION

The authors received no specific funding for this study.

## CONFLICT OF INTEREST STATEMENT

E.H. received lecture fees from Taiho, and E.H. and T.I. received research expenses, scholarship donations (grants) from Taiho. H.B. and H.N. are editorial board members of the *Annals of Gastroenterological Surgery*.

## ETHICS STATEMENT

Approval of the research protocol: The study protocols of KHBO1401 and KHBO1401‐3C were approved by a suitably constituted Ethics Committee of each participating institution and conform to the provisions of the Declaration of Helsinki; Committee of Hyogo Medical University, Approval No 202204‐251.

Informed Consent: Before the study began, written informed consent was obtained from all the patients in KHBO 1401 study.

Registry and the Registration No. of the study/trial: KHBO1401 (ClinicalTrials.gov, NCT02182778 and the UMIN Clinical Trials Registry, ID 000014371).

Animal Studies: N/A.

## Supporting information


Figure S1
