# Supplementary material for: Impact of conversion surgery after chemotherapy in patients with initially unresectable and recurrent biliary tract cancer
Source: Ann Gastroenterol Surg. 2023 Jul 19;7(6):1009–20. doi: 10.1002/ags3.12713 (PMC10623972; doi:10.1002/ags3.12713)
Supplement: Supplementary file 1 — Figure S1 [file AGS3-7-1009-s001.docx]

Supplementary Figure 1a

Supplementary Figure 1b
